# Proceedings of Eighth Annual Session I. H. A.

**Published:** 1887-07

**Authors:** 


### THE EIGHTH ANNUAL SESSION OF THE INTERNATIONAL HAHNEMANNIAN ASSOCIATION.

The International Hahnemannian Association met at Long Branch, N. J., June 21st, 22d, and 23d. Among those participating in the discussions, which were most interesting, were Drs. H. C. Allen (of the *Advance*), J. V. Allen, E. A. Ballard (our most useful Secretary), B. L. B. Baylies, J. B. Bell, J. A. Biegler (our indispensable chairman of Board of Censors), T. L. Brown, C. W. Butler, Alice B. Campbell, G. H. Clark, Stuart Close, J. B. G. Custis, W. S. Gee, W. J. Guernsey, A. Harvey, W. A. Hawley, Harlyn Hitchcock, E. P. Hussey, J. T. Kent, S. A. Kimball, C. H. Lawton, Ad. Lippe, Samuel Long, J. F. Miller, E. B. Nash, W. A. D. Pierce, Mahlon Preston, Franklin Powel, E. Rushmore, E. W. Sawyer, Julius Schmitt, S. Seward, J. W. Thomson, P. P. Wells, Wm. P. Wesselhœft.

Besides these physicians, members of the Association, there were present delegates from several societies and some lady visitors, who were most welcome and who contributed not a little to the success and the interest of the session. May they come again next session and in greater numbers. The meeting was called to order promptly at eleven A. M. by the President, Dr. J. T. Kent, who delivered a forcible and suggestive address, which was listened to with marked attention and interest. This address will be found in another part of this issue of our journal; heed its suggestions. Next in order came the report of the Secretary, who had several important suggestions to make.

Drs. E. Rushmore and E. W. Sawyer reported as delegates for the New Jersey and Indiana State Societies; both reported that pure Homœopathy was on the increase in their States. Dr. Paul C. Grant represented the Rochester Hahnemann Society. A letter was read from Dr. John Hall, referring to the need of a new and better translation of the *Organon*. Referred to a committee, consisting of Drs. Wells, Lippe, and Wesselhœft, to report at next meeting. The Committee on Revision of the By-Laws reported through its chairman, Dr. Wesselhœft. The By-Laws as adopted are as follows:

SEC. 1. This Association shall meet annually at such time and place as may be determined by a majority of the members voting.

SEC. 2. Its officers shall be elected annually by ballot.

SEC. 3. Applications for membership may be received at any regular meeting; they shall be indorsed by three members of the Association who are in good standing. Said indorsement shall be made, not upon the general reputation of the applicant, but upon the positive knowledge of the integrity of the applicant's practice on the part of at least one of the indorsers. It shall be the duty of the chairman of the Board of Censors to send the names of all applicants to the Association at least a month before the next annual meeting.

Sec. 4. The application shall be in the possession of the chairman of the Board of Censors for the period of six months and the applicant shall place in the hands of the chairman, at least three months before the end of the year, an original thesis consisting either of an original proving or a clinical report of three cases treated by him or her. If the applicant be elected, his or her thesis shall be referred to the Committee of Publication; if rejected, the thesis shall be returned to him or her.

Sec. 5. At the next annual meeting after applications have been received, and upon the recommendation of the chairman of the Board of Censors, an election by ballot shall be held, and a two-thirds majority of the members present shall be necessary to elect. If an applicant be not elected he or she may, upon a majority vote, if he or she desires, receive a second ballot at the next regular meeting.

Sec. 6. The name of any applicant for membership in this Association which has been rejected by an unanimous vote of the Board of Censors shall not be presented to the Association for action in any case; but in case of the rejection of any applicant by a majority of the votes of the Board of Censors the minority of that Board may report the name of such applicant to the Association for final action.

Sec. 7. The annual dues of this Association shall be five dollars, payable in advance.

Sec. 8. Any member who shall fail to pay his annual dues, shall, for the time during which they remain unpaid, forfeit all privilege of membership, except by unanimous consent of members present.

Sec. 9. The Constitution and By-Laws may be amended at any annual meeting of the Association by a two-third vote of the members present, notice having been given in writing at a previous meeting

Sec. 10. At each annual meeting of this Association the President shall appoint a chairman of each of the following bureaux: Materia Medica, Clinical Medicine, Therapeutic Surgery, Obstetrics, and Diseases of Women and Children.

Sec. 11. Inasmuch as this Association is international in its character, that for all officers foreign members be permitted to vote by proxy.

The Board of Censors then reported, and the following were elected members: Dr. W. W. Butler, Brooklyn; Dr. George H. Carr, Galesburg, Ill.; Dr. R. L. Thurston, Brooklyn; Dr. B. L. B. Baylies, Brooklyn; Dr. E. T. Adams, Toronto; Dr. Harriet H. Cobb, Cambridgeport, Mass.; Dr. H. P. Holmes, Sycamore, Ill.; Dr. Charles E. Chase, Utica, N. Y.; Dr. T. S. Keith, Newton, Mass.; Dr. Flora A. Waddell, Wauseon, Ohio; Dr. J. D. Tyrrell, Toronto; Dr. S. L. Eaton, E. Orange, N. J.; Dr. W. J. H. Emory, Toronto; Dr. M. P. Wheeler, Boston; Dr. J. W. Thomson, N. Y.; Dr. J. V. Allen, Philadelphia; Dr. D. H. Riggs, Washington; Dr. J. U. Woods, Holyoke, Mass.; Dr. Frank Powel, Chester, Pa.; Dr. F. C. Hood, Marysville, Cal.; Dr. Nathan Cash, Uhrichsville, Ohio; Dr. W. H. Baker, Rochester.

The next business in order was the report of the Bureau of Materia Medica.

Dr. Wesselhœft, the chairman, said: "I will open this Bureau with a thoughtful and very important paper by Dr. Lippe, upon 'A Progressive Materia Medica: How it is to be Developed.'" After Dr. Lippe had read his paper, there followed one by Dr. P. P. Wells, on "Errors in Drug Proving" (which paper we will soon publish in full). Dr. Wells' paper was listened to with marked attention, and was discussed by Drs. Lippe, Hitchcock, Nash, Ballard, Gee, Hawley, and others.

A paper on Lac caninum was submitted by Dr. E. W. Berridge, London.

Dr. H. Hitchcock read a paper upon "Homœopathy and its Relations to the Germ Theory"—a most interesting paper. The next paper was read by Dr. E. B. Nash on the "Tissue Remedies." Much discussion ensued, chiefly upon the use of these remedies. Concerning Magnesia phos., Dr. Wesselhœft said: "I made a cure with Mag-phos. very similar to the one Dr. Nash has just reported. It is one, I think, could never have been made without Mag-phos.$^{cm}$. It was an astonishing cure. The case was of a neuralgic character; the patient was an old lady of sixty-six years. I saw her for the first time about six years ago. I wondered that any cure could be made upon any one who was so attenuated, thin, and so lacking in vitality. I have observed that Mag-phos. had three peculiarities. It is an entirely right-sided remedy, pains shifting, supra-orbital, and relieved by warmth."

Dr. Nash's paper was further discussed by Drs. Lippe, Baylies, Wells, Ballard, Allen, Nash, and others.

A long and profitless discussion was held upon the question of publishing the transactions. After much debate they were referred to a committee for publication, consisting of Drs. Wesselhœft, Butler, and Ballard. A motion was made that the Society publish their proceedings in journal form, the journal to be published by the Association, but this was considered inexpedient at present.

Dr. S. Swan's resignation was received and promptly accepted.

The consideration of report of the Bureau of Materia Medica was resumed; papers presented by Dr. S. A. Kimball, a repertory on gonorrhœa and balanorrhœa (one of the most useful contributions yet made to our literature by the Association); Dr. George H. Clark, "A Few Points on Dulcamara;" some "Verifications," by Dr. J. A. Biegler; a partial proving of Salicylic acid, and also one of Mellilotus off., by Dr. H. C. Allen; a proving of Sanicula spring water, by Dr. Sherbino; a proving of Dulcamara, by Dr. Wesselhœft. It will be seen, even from our meagre report, how numerous and valuable were the papers presented by the *Bureau of Materia Medica and Provings*. It is the most important bureau of the Association, and its work is well done. Such work from year to year cannot fail to accomplish much good.

The next bureau to report was that of surgery, Dr. Edmund Carleton, chairman. Unfortunately Dr. Carleton was detained at home by sickness, but his place as chairman was ably filled by Dr. J. B. Bell. The first paper read was the history of a mammoth ovarian tumor removed by Dr. Carleton, and now reported by him. The report showed how homœopathic prescribing helps the surgeon in securing recoveries after severe operations. After some discussion of Dr. Carleton's paper, one by Dr. Julia Morton Plummer was read by the lady herself. The subject of the paper was "Antisepticism;" the thesis was exceedingly well written and unusually well delivered. Dr. Plummer's paper was received with much gratification. Dr. J. N. Lowe read a paper upon "Incised Wound of Knee-Joint," which was discussed by Drs. Rushmore, Gee, Sawyer, and Brown. Dr. Sawyer spoke of his use of dog-wood locally to prevent gangrene, and which he claimed it did.

A paper was read showing the great evils which follow the suppression of gonorrhœa and syphilis. The exceedingly interesting discussion which followed we hope to publish in full later.

After the Bureau of Surgery had reported, the Association received a delegation from the Women's Homœopathic Hospital

Association, of Pennsylvania. A report of their work was read by Miss Ramborger, showing the great difficulties they had to encounter and the useful work accomplished. Their hospital is now built and will be formally opened in the fall. The Association listened to the report with interest and formally expressed its commendation of the work these ladies are doing.

The Bureau of Clinical Medicine reported through its chairman, Dr. E. W. Sawyer. First papers read were two by Dr. G. H. Clark; Dr. J. A. Biegler read a number of short and interesting papers. One of Dr. Biegler's papers gave the history of a case of "Hay Fever," which elicited a long and very interesting discussion, that we will give in full later. Dr. W. S. Gee next read a paper on "Clinical Verifications," which was full of good points and was thoroughly discussed by Drs. Nash, Baylies, Gee, Rushmore, Brown, and others. Dr. C. W. Butler read a very instructive paper, giving a clinical study of Sulphuric acid. The value of Sulph-acid in mechanical injuries was described; Dr. Sawyer cautioned against its use "low" in lung troubles, saying he had seen hemorrhage follow such exhibition. Dr. Schmitt alluded to a case of scrofulous opthalmia he had cured with Sulph-acid, his attention being drawn to the remedy by the blue spots on the patient's hands. Dr. Lee called attention to the use of Aconite and Sulph-acid in mechanical injuries of the eye, as from sparks, etc. Dr. Lippe remarked that Symphytum was *the* remedy for injuries to the eye from blows, etc.

Dr. E. Rushmore also read some instructive "Clinical Notes." One gave the history of a case of intermittent fever, which drew some interesting talk upon that troublesome disease. Dr. Hitchcock asked why Dr. Rushmore gave in one case three powders twelve hours apart, and in another case three powders an hour apart? Why not let one dose act?

Dr. Rushmore—With reference to the prescription made in the case of intermittent fever, three powders twelve hours apart, it was nearly in accordance with the suggestion for proceeding in such prescriptions made to me some years ago by our venerated friend, Dr. Lippe, that the way to administer in such cases was to give one dose shortly after the decline of the paroxysm, and another about three hours before the expected recurrence, and knowing the time of beginning, and the time at which the third dose would terminate would be a few hours before the expected recurrence, it was ordered to be given in that way. One hardly knows sometimes why he repeated, and why he happened to give two or three doses in one day. The patient was suffering acutely where the three doses were three hours apart. The sufferings were of that intense kind where the pressure is to give medicine until relief is seen.

Dr. Lippe—Hahnemann tells you to give the remedy in intermittent fever either two hours after the paroxysm is over or two hours before the next paroxysm is expected, and you may use two doses without doing any harm—two hours after the paroxysm is finished and two hours before he expects the next paroxysm. That is according to Hahnemann's advice.

Dr. Ballard—If I may I would like to say a word, without being out of order, on that repetition.

The President—Yes, sir; that is in order.

Dr. Ballard—I would say in regard to the repetition of the remedy in intermittent fever that I have not found that advice very good with me. I will just cite a case to show where the repetition of a remedy in intermittent fever may do a great deal of harm. The case was evidently one of Arsenicum. I came in the evening just as she was through with the last stage. There was no sweating, and I was told she was coming out of the fever. I gave a dose of the two-hundredths. She was having the attacks every alternate day. I left the dose for the following morning and for the next evening. That was some years ago, before I handled them quite as carefully as I do now. The next forenoon she had a slight chill, a very unexpected one, and they came to me and asked my advice about that; and I advised not to take the third powder. So two powders only were taken. On the next morning I was to be sent for if she had a chill. She had one about eleven o'clock, and when I entered the room I found her rattling pretty lively. She said to me: "Dr. Ballard, I will give you one more trial and then I will take my Quinine. I will not stand this any longer." I told her that was the last chill she would have. I could see that was the hardest one and that I had produced an effect with the remedy. On the next day when the chill was due she had just the slightest appearance of a chilly sensation, and that was all she had. That taught me a lesson in regard to giving Arsenic in chills, and in regard to the repetition of a dose in chills. So that now I take my case, being very careful to know what I prescribe. I do not prescribe at all until I know pretty thoroughly what I am to give, and then I give one dose, and that is all I do give, and I do not repeat it until I am certain that another remedy is required.

Dr. Lippe—That is exactly my practice now; Hahnemann did advise giving the remedy during the apyrexia, and never during the attack; he then advised them at that time to give it right after the paroxysm was over, and probably give another dose before the next paroxysm was expected.

Dr. Gee—I would like to ask what potency Hahnemann used at that time.

Dr. Lippe—There is no doubt that there has been a change in the use of potencies. We have progressed, and we give a higher potency, and this higher potency will not stand a repetition. That is the difference. The dose you give now after a paroxysm is over, in a high potency will act powerfully. It does not require a repetition. At Hahnemann's time it did. So Hahnemann advised correctly at that time. But our experience now teaches us that we are going forward all the time and not backward.

Dr. Ballard—Mr. President, there is one little point I would like to ask the experience of others about, and some advice. I do not know who it is, but some one advises giving a dose immediately after a chill, before the fever has come on; if there is any intermission whatever to give the indicated remedy at the end of the chill and let it alone. I have tried that in a few instances, but I don't like it, as I prescribed it for a little boy at one time, thinking Apis was the indicated remedy. I gave a dose, a 52M, to be taken immediately after the next chill, and it was given, and that poor little fellow rolled around the floor, and he could not have anything on him, and his mother said he was broken out with bee stings from the crown of his head to the soles of his feet, and that he suffered most horribly all night, and I have not given another dose of Apis at the termination of the chill.

The President—If you will pardon me for speaking from the chair, I would like to say in regard to these intermittent attacks in the West—and I think that we know about intermittent fever, and they are of the highest type of the periodical paroxysmal troubles, and we have had a good deal of experience with them—that my practice is to give one dose of medicine when the paroxysm has entirely finished, but never to administer a dose of medicine preceding a paroxysm. If I am called to a case of intermittent fever, and the paroxysm is soon to come on, I give Sac. lac., and when the entire paroxysm has finished the dose of medicine is administered. If you do not observe this practice you will fail in a very large number of cases. I am speaking now of the intermittent fever of the West. You will be puzzled, and you do not know why. If you give the medicine from one to three hours before the paroxysm you will have the most severe paroxysm as a result of your medicine. It seems to add fuel to the fire. This has been my experience. Dr. Allen has seen a good many of these Western fevers, and I

would not be at all surprised if he could bear me out in this experience. Dr. Allen, what have you to say?

Dr. H. C. Allen—Since I have begun to use potencies above the thirtieth my experience emphatically is that the remedy should not be given until after a complete cessation of the paroxysm. I have had the best results when I have followed this mode of prescribing. I remember distinctly a case that came down to our hospital from the Saginaw Valley—a case of chills that had lasted for nine years. One of my colleagues whom he came to consult for a surgical affection heard of the case and learned that it was one of intermittent fever. He went to the boys and said, "I will show you how to cure this. I can cure him," and he made a prescription of ten grains of Quinine three times a day. After treating the patient for a number of days, and the patient assuring him that he had taken a half bushel or more of Quinine in nine years, he told me one morning as he went into the hospital that there was a case of chills over there that I had better go and look after. So I took a number of our Senior Class and two of my assistants and went. I found a very peculiar case, and one you rarely meet with. The chills began promptly at half-past five every afternoon, and lasted for an hour. They were very severe, always beginning in the thigh from the knee to the hip, and from there extending over the body. He would sit with his legs on each side of the register and remain until the chill had passed away. Then the fever came on, lasting for several hours, and it was followed by a profuse, drenching perspiration, which ended about two or three o'clock in the morning, even wetting the bed. I suggested that a few doses of Thuja would finish that case without any difficulty, and he received two doses of Thuja $^{200}$ and never had another chill.

Dr. Sawyer—Why two doses?

Dr. Allen—I was in the hospital and I could not do exactly as I should in private practice, but I may say that hospital practice is a nuisance in the practice of Homœopathy as far as I am concerned. That is my experience. There are too many cooks, and a great many cooks often spoil the broth. The one pursued generally is to give one dose and leave another dose, to be given if certain circumstances occur. Whether they arose or not I could not say, but through the mistake of the boys or the assistants he had two doses. That is the way it happened, I think.

Dr. Biegler then related some very interesting experiences which he had while in charge of the Quartermaster's Hospital while in the Department of the Gulf.

An instructive paper by Dr. John Hall, Sr., on acute diseases in horses was presented, which will soon be published in full in these columns. Papers were presented by Dr. William Jefferson Guernsey, on variola; by Dr. J. N. Lowe, upon clinical experiences; by Dr. W. H. Baker, on warts; by Dr. E. W. Sawyer, on Lachesis in a case of diphtheria, and one on "ague," cured by one dose of Pulsatilla.

The last bureau to report was that on diseases of women and children, Dr. H. P. Hussey, chairman. The first paper read was by Dr. Lawton, entitled "Dynamic Disturbances of the Puerperal State;" the paper was very instructive and interesting, as are all of Dr. Lawton's.

Dr. Ballard read a paper giving clinical hints for prescribing in parturition.

Dr. Schmitt remarked that he gave Pulsatilla when the woman cries "Oh! my back! Oh! my back," like Causticum, but there is no exhaustion. There is no debility in the case. She has not been sick before, the pains do not press down, and the pains are in the buttocks, like Kali carb., and she wants to have her back pressed. I have given her, in this condition, Pulsatilla, and have always found a response. At the same time I want to mention two cases that happened to me lately. Sepia $^{200}$ was given in one case, before the uterus had fully opened. It was about half opened, but the pains were not sufficient to open it entirely, and there was tenderness of the anterior lip of the mouth of the uterus and the pain above the pubes, as if everything would come out. In both cases these same conditions prevailed. In one case I had to give a dose of Sepia $^{200}$ to produce an effect, but in the other case after one dose of Sepia $^{mm}$ the mouth opened readily and the membranes ruptured. From that time there was no pain, but the woman described the feeling as if she were at stool, and persistently asked me if she had not had a passage. She stated that it was a bearing down. There was no single pain after that, but the child was born without pain almost in the natural condition which we ought to expect. Sepia was the remedy given.

Dr. Biegler—I would like to say one word. I, like Dr. Schmitt and others, have had grand results from the administration of remedies in labor, but I must confess that my last case was rather a damper upon all the rest, while I have had other illustrations in the same way. I do not mean to discredit the efficacy of remedies, because I know they do change the labor and change the character and direction of the pain. I know that. I think as an illustration of my last case. It was a case where

I found the os turned and apparently of the size of a quarter, but the edge of it was spread out then as thin as a piece of tissue paper from the head. It was a vertex presentation. It was a case which I usually expected time to remedy, and I simply said to the lady, " You are all right. I will go down-stairs and pass away the time. You take a little time." I put on my cuffs, and I thought before going down whether to pick out anything. As I stood talking to her, looking at my case, she at once exclaimed: " O Doctor! come!" and there was the head in the bed at once. Now, if I had given that woman only a moment before a certain medicine it would have been a grand result for me, and I could have given the remedy the credit.

Dr. Hawley—Mr. President, I had just about that thought in the Doctor's first case. The woman was crying and she wanted relief, and the thought had occurred to me to allow time, when the pains came on again. I did not give Aconite or anything else.

Dr. Hussey—That is a thing I have occasionally seen happen. I have explained it to myself and conceded that the cause might be possibly that the pains which cause so much mental disturbance and are so aggravating are merely contracting the circular fibres of the uterus, which do not forward matters any. They simply produce pain in the back, a very painful sensation, and then expulsion will come on and progress is made, and then the crying is over, the child is progressing, and labor will soon terminate. That is liable to occur at any time.

Dr. Ballard—Mr. President, these cases were presented simply because these symptoms were well marked symptoms which belong to the remedy. I have seen them spoken of repeatedly. I did not notice it until this case, however. That lady had been thrashing around the bed for a long time, and the restlessness was very great, more than what usually occurs, and you could not keep her covered up. You could not keep her in any part of the bed, and the agony that was expressed clearly indicated the mental disturbance which calls for Aconite. She said she felt as if a large cannon ball had entered and crowded everything down and was pushing everything out and giving relief to all her sufferings.

Dr. Hawley—Mr. President, I think I often spoil a mighty good case by my not letting it alone. In these cases there is no sickness but simply a natural, orderly fulfilling of the functions of nature. I had a case a few weeks ago that will illustrate this. My daughter-in-law in her first labor had an exceedingly difficult labor, so that she was in constant agony and distress for thirty-six hours. I did not attend her. She was not with me. She is a delicate and rather feeble woman and decidedly scrofulous in habit, and a year ago last April her husband brought her to his father's house because she was so feeble. My first remark to my wife was, after I saw her, that I would not give two cents for her, that she would go right down with consumption. She had a terrible cough and a hectic appearance of her face. However, she soon got over that and got much better. The remedies under which she improved were Phosphorus 80M potency (Finke), single dose, and being constantly out-of-doors. She had been necessarily confined in the house pretty much since the birth of the child, which was then two years and a half old, and I took her in my carriage every day. She was out with me every day, rain or shine, snow or blow. Soon, very much to her surprise and regret, she found herself pregnant again. I continued to keep her out-of-doors every day when I was in town during the winter. On the first day of April last she called me at five o'clock in the morning saying she had a new experience. She was having a little discharge of water. I told her I would send her mother up to her, and she would probably fix her better for that than the doctor could, and I went down-stairs and went to bed again. My wife went up and administered to her comfort and returned to her bed herself. All day long she had a little trouble of water but no pain. She busied herself about the house, as she did not care to go out to ride with me that day. When I came home that night she had gone to bed and I went up to see her. I found her comfortable without any pains, and so went to bed about as usual. The nurse was with her, and I told her she might call me if she needed me. The call came for me, so that I went up to her room at exactly twenty minutes past eleven o'clock that night, and just twenty minutes before twelve o'clock I had the baby in my hands. Well, now, if I had only given that woman something during all those nine months to insure an easy labor what a pretty story I could have told, but I did not give her anything.

Dr. Schmitt—You gave her Phosphorus?

Dr. Hawley—I gave her Phosphorus, but that was months before she was pregnant. I did not give her any medicine, and it was nothing but the natural process.

Dr. Allen—Do I understand Dr. Hawley to say that Phosphorus is not a medicine?

Dr. Hawley—I do not think that Phosphorus had anything to do with it.

Dr. Allen—But still you gave her Phosphorus?

Dr. Hawley—That was months before she was pregnant, but I have no doubt her restoration to health had something to do with her labor—I have no question about that—but how much I could not say. She got plenty of exercise out-of-doors in the air and I have no doubt that did her a great deal of good.

Dr. Biegler—Mr. President, Dr. Hawley's case of the administration of Phosphorus several months before pregnancy reminds me of an occurrence which has given me a reputation in certain part of the country. A lady came to me who was racked with the aphthous state throughout the whole system, lips, mouth, and the vagina—the external part. She was a wreck from dosing and from being worn out by want of nutrition and all that. I prescribed for her, and was getting her well. In the period of a few months I had made her two or three prescriptions, when all at once after she had got pretty nearly well, but not quite, to my surprise and astonishment she announced that she was pregnant. I was satisfied that she had been cured, for she never could have become so otherwise. But the joke of it is she went home and told the ladies to keep away from me, that I was a dangerous fellow. (Laughter.)

Dr. Schmitt read a paper narrating a case of anemia cured with Ferrum; Dr. Hussey read a paper on "Whooping Cough," showing clearly the power of homœopathic prescriptions to cure and to shorten the attacks of that disease.

Dr. Rushmore suggested that the chairmen of the several bureaux arrange their bureaux so as to include as many as possible of the younger members of the Association and also the new members. Dr. Hawley remarked that an attempt should be made to secure papers from the foreign members of the Association. The bureaux should be announced promptly and so that the work for the next meeting be commenced at once. Hard work will be needed to make the session of 1888 more profitable and more interesting than the one just closed.

The President—Before we adjourn I would like to ask Dr. Lippe for his blessing. We will not meet again for a year. We ask you for any such remarks or kind advice that you would like to offer to the Association.

Dr. Lippe—I can only say this much; I have attended a great many Homœopathic Conventions, but I seldom stayed them out; that this meeting has been a most satisfactory one—the most satisfactory one that I have ever attended in all my life. [Applause.] This Association has done more in the last two years than the American Institute has done in the last quarter of a century, a great deal more in advancing Homœopathy than they could do in a quarter of a century to destroy it. I think that we have got ahead of them now. We are organized. We never were organized until we organized seven years ago in Milwaukee. We are now increasing in numbers, increasing in earnestness, increasing in importance of labor, and there is no doubt that in course of time this Association will be the only homœopathic institution in America, and the Institute will sink down into nothing. I expect nothing else; they will lose their numeric strength and they must adopt the name of Eclectics. They will be compelled to do that by public opinion and by the opinion of medical men in America, and the moment they drop the name then the organization falls. They have been false to Homœopathy. The American Institute is not homœopathic; we have the only Hahnemannian Institution. And the Hahnemannian colleges and the Hahnemannian journals and Hahnemannian societies are all sailing under false colors. They will have to strike those colors, and this Society will compel them by moral influence, and by nothing else, by the moral influence which we exercise over them, to strike those colors, and then I say we shall be left with our colors flying over the Society as the only Homœopathic Society in the United States. [Applause.]

Dr. Ballard—In my capacity as Secretary during the last year it has been my pleasure to observe, perhaps more than most of the other members, the indications which have been no doubt growing brighter, year by year, of the progress and the strength of our school, and I mean by our school that portion of it which we represent. I have received inquiries from young men, very young practitioners, from various parts, inquiring about the Association, the terms of membership; they can see necessities which exist for such an association for the good of the cause. They are casting about them, and many practitioners are writing to me to inquire about how they shall get into the broad and clear highway leading on to the truth. In my own city I am sorry to say that the good which this Association is doing is really and truly needed. Only a few months ago a graduate of one of their colleges there came in and introduced himself and inquired if I was a member of the I. H. A. He said he understood that I was, and that would indicate that I was a homœopath. He had been graduated by a homœopathic college a year before, and dropped over into the mud clear up to his ears. He said, "I am wallowing and wallowing, and I don't know hardly how I can get out of it. I don't know one word about Homœopathy now, though I have been graduated from their school. I do not know any more than I would if I was a graduate of the old school." I told him he was just the kind of material we wanted, because he was conscientious. But I urged him to go on. So it is seen on every hand, the good that this Association is doing, and if we can have a few more such meetings as the last one and this one, I do not think that any great length of time will pass before we shall be in a position where the American Institute may well take the back seat. Numbers do not always count. Now, Mr. President, I want to thank the members, each and all, for the courtesy which they have shown me in my endeavors to perform the duties of my office, and they have by that courtesy made those duties duties of pleasure. I certainly have enjoyed myself at this Convention most unusually, and I can thank each and every member for his good heart, courtesy, and kindness.

Dr. Lee—Mr. President, may I add one word? In his address in 1881, at Coney Island, Dr. Wells, I think, told us upon what lines we would achieve success, and that we have done. I would like to read a few lines, if in order. After outlining the principles of this Association and describing some work which he thought we should do, Dr. Wells says: "Work of this sort, persisted in, will by and by mature a power greater than any argument, however masterly, or than any controversy, no matter with what earnestness it may be waged. Work of this sort will in time, by its results, so demonstrate to the public mind the superiority of the pure practice of Homœopathy we advocate over that which is partial or mixed, as well as over that of the old school, that these gentlemen, recognizing the education the public has thus received, and at the same time the confidence of the public they themselves have lost, are not in the least danger of neglecting to make haste to claim their share of the honor a numbering with those thus diligent is sure to confer. Thus, and only thus, can the interests of true Homœopathy be advanced and the objects for which this Association was organized be secured." I think Dr. Wells' advice was sound; we have followed it and *have* secured a great influence for good. Simply by doing good work we have secured our present position, and our influence *is* doing good work.

Dr. Hawley—I want to coincide in that expression. It is only by just such work that Homœopathy has ever made any progress, and the hitch which has been made in the progress of Homœopathy has come of the fact that many so-called homœopathic physicians have been chasing that will-o'-the-wisp called Pathology, instead of giving their attention to the light of the law of Similia similibus curantur. It is certainly true, in my neighborhood, at least, that there is a great reaction in favor of fidelity to the law. My own neighbors, who have been so imbued with the spirit of mongrelism that they never would speak of me, for instance, in any family where they went but as a high potency man, are beginning to come around and ask how it is that these things are done, and to express a wish that they could do it. Perhaps by and by that wish will become strong enough in them, so that they will begin to study; no one of them does study that I know of now.

Dr. Ballard—I move that we adjourn *sine die.*

Dr. Lee—I move that we have an address by the President and have his blessing.

Dr. Ballard—I withdraw my motion in favor of the President's blessing.

The President—Gentlemen, I simply thank you away down to the bottom of my chest—and it is a big one, you know—for the manner in which you have assisted me in presiding over these meetings. I certainly have had no serious tasks, but a very pleasant duty. I have listened to the discussions with great reluctance, because I wanted to take a part myself. I have regretted very much that I have been tied down to this chair by custom. It was said when I was elected to the position of president that they had put me where I would have to keep still, so I have tried very hard to keep still, and we have listened to the wise counsel of Dr. Wells and Dr. Lippe, who have watched the growth and development, not simply of the I. H. A., but of Homœopathy in the United States and in the world. Homœopathy has passed through many vicissitudes, wonderful discouragements, ups and downs, and until finally this Association was organized. You have seen its growth and its development, until now it seems that we are upon a permanent footing. It was not without a great struggle, and not without evil possibilities. One thing I believe to be most certain, that should any calamity come over us that should destroy this I. H. A. it will be fifty years before we have another Homœopathic Association. This leads me to say to you that we should watch every corner, that we should watch all our weaknesses, that we may work together unanimously and harmoniously for the development and growth of this Association. It is the only general society where homœopathists can labor. We have local associations; they are small, and not very useful because of their limited number, but this Association is growing. Its possibilities are wonderful, if it make no mistakes. There is one neglect that I

think we have to look after in this Association. We should have a Bureau of Philosophy. [Applause.] We ought to have a bureau devoted to the *Organon* of Samuel Hahnemann. We have a Bureau of Materia Medica and of Clinical Medicine, which really are the practical outcome of our philosophy; they are the means by which we administer our philosophy; but we ought to have a bureau in which shall be studied at least one paragraph of Hahnemann's *Organon* in the early portion of every session. It should never be omitted. It should be followed out regularly and be a part of the work. This bureau is in relation to the development, the enlargement, and the sentiment of Homœopathy. I hope that at some future time you will have a Bureau of the *Organon*. Let it simply be a Bureau of Homœopathic Philosophy based upon the *Organon* of Samuel Hahnemann. I cannot begin to express my real thanks for what I have seen and heard and felt at this meeting. It certainly is the greatest meeting, I believe, in the history of this Association, and there is no reason why the next meeting should not be even greater than this; but remember that it is not so much the increase of members, but the more we come together the more we become acquainted with each other, and the more we work with each other, so do we become more useful to each other. I thank you from the bottom of my heart for the kind feelings which I have always received in this Association.

Dr. Ballard—I will offer the standing resolution, which I will write out, that we add to the number of our bureaux one of Homœopathic Philosophy based on the *Organon*, which shall take precedence of all other bureaux. You can confine it to any number you choose. I think it would be just as well to make up, say, three or four sections—have three or four members on that bureau—three, I think, would be enough.

Dr. Biegler—Some sections are of very great length. In our local Association we have the *Organon* read at every meeting.

Dr. Allen—I think we ought to shorten the title. I was going to make a motion, but Dr. Ballard has made one, and I think we should never put to the next session what we ought to do this year and what we can do this year, hence I second the motion of Dr. Ballard, and I think that the title would be tautological—Philosophy of Homœopathy could not be based on anything. The title Philosophy of Homœopathy is good enough. While I am on the floor I would like to suggest also that Dr. Lippe be made the chairman of the bureau for this year.

The President—It has been moved and seconded that a Bureau of Philosophy of Homœopathy be instituted in our regular curriculum.

Dr. Hawley—I want to say that I most heartily approve of the creation of such a bureau, and I want to tell you one reason why I approve it. In our little Association in Central New York we found a very curious result from the practical institution of such a bureau. We got rid of all the mongrels. Now if we do this thing in this Association, and one of those fellows ever gets in here, he won't stand it two sessions.

The question being on the resolution of Dr. Ballard, it was agreed to, and the Bureau of Homœopathic Philosophy was created, with Dr. Lippe as first chairman.

Dr. Gee—I do not feel it just on my part to leave without an expression of thankfulness and gratefulness as a younger member of the Association, one who is in a modest way trying to help others as well in this work, which is uphill in the extreme, but I have universally had from the older members of this Association and profession the kindest help in the way of letters, and I feel grateful for this, and I hope that this brotherly feeling, this helping hand, will be continued and be extended to us who feel the need of it—who feel the need of that sympathy—for we are alone at times, acting upon our own responsibility, and I am sure a helping hand will be of great satisfaction to our younger members of the profession in their coming in. It is not an easy task to step out alone, and the help from our fathers is appreciated more than can possibly be told.

The following are the officers and chairmen of bureaux for 1886:

President, Wm. P. Wesselhœft, M. D., Boston.

Vice-President, C. W. Butler, M. D., Montclair, N. J.

Secretary, E. A. Ballard, M. D., Chicago.

Treasurer, W. A. Hawley, M. D., Rochester, N. Y.

Censors, Dr. J. A. Biegler, *Chairman*; Drs. W. S. Gee, E. Rushmore, C. W. Butler, and J. B. Bell.

Committee on Publications, Drs. Wesselhœft, Butler, and Ballard.

Necrologist, Dr. T. Dwight Stowe.

Bureau of Homœopathic Philosophy, Ad. Lippe, M. D., Chairman.

Bureau of Materia Medica, Dr. W. S. Gee, Chairman.

Bureau of Surgery, Dr. J. B. Bell, Chairman.

Bureau of Clinical Medicine, Dr. H. Hitchcock, Chairman.

Bureau of Obstetrics, Diseases of Women and Children, E. P. Hussey, Chairman.

Next meeting to be held at Niagara Falls; time to be announced later by Executive Committee.

We would suggest that the Association adopt Cushing's or some other manual of parliamentary rules to guide them in their discussions. Much valuable time might be saved by a little strict ruling on the part of the Chair.

So closes our brief report of the eighth session of the I. H. A. The meeting was a grand one. May each succeeding meeting be better and larger till nothing be left to be desired!

