# Correction

**Published:** 1887-10

**Authors:** 


Correction.—July number, page 260, line 15 from top, should read thus: Dr. Schmitt remarked that he gave Pulsatilla when the woman cries, "Oh! my back! Oh! my back!" like Causticum, but there is not the exhaustion from previous sickness as under Causticum. She wants her back pressed like Kali-carb., but the pains go from back to front, not down the buttocks, as under Kali-carb.

